# Supplementary material for: Citizen Science for Mining the Biomedical Literature
Source: Citiz Sci. Author manuscript; Available in PMC 2018 Nov 9. (PMC6226017; doi:10.5334/cstp.56)
Supplement: Supplemental Figures [file NIHMS992547-supplement-Supplemental_Figures.pdf]

Supplementary figures

Figure S1

Let's start by marking diseases

Mark all the diseases in the sentence below.

> Does choice of insulin regimen really matter in the management of diabetes ?

Sometimes you will see multiple instances of the same disease - Be sure to mark them all!

Mark all the diseases in the sentence below.

> To assess the management of diabetes , we reviewed records of 20 diabetes patients .

Sometimes the disease is described by a conjuction of several words. Mark these disease conjunctions as a single span of text.

Mark all the diseases in the sentence below.

> Of the 20 patients , 17 had type 2 diabetes mellitus .

Sometimes the disease conjunctions are separated by words like 'and/or'. Decide if there are two distinct diseases to highlight, or if it is a single disease conjunction.

Mark all the diseases in the sentence below.

> The remaining 3 had inherited and/or type I diabetes mellitus .

Sometimes different diseases are discussed. Mark all the diseases below.

Remember to mark disease conjunctions as spans and to mark different diseases separately.

> Of the 20 patients , 10 patients were also diagnosed with heart disease or rheumatoid arthritis .

Sometimes the diseases are abbreviated. Mark all instances of disease abbreviations.

Mark all diseases in the sentence below.

> We will discuss the effect of different insulin regimen on type 2 diabetes mellitus patients (ie- T2DM patients) ...

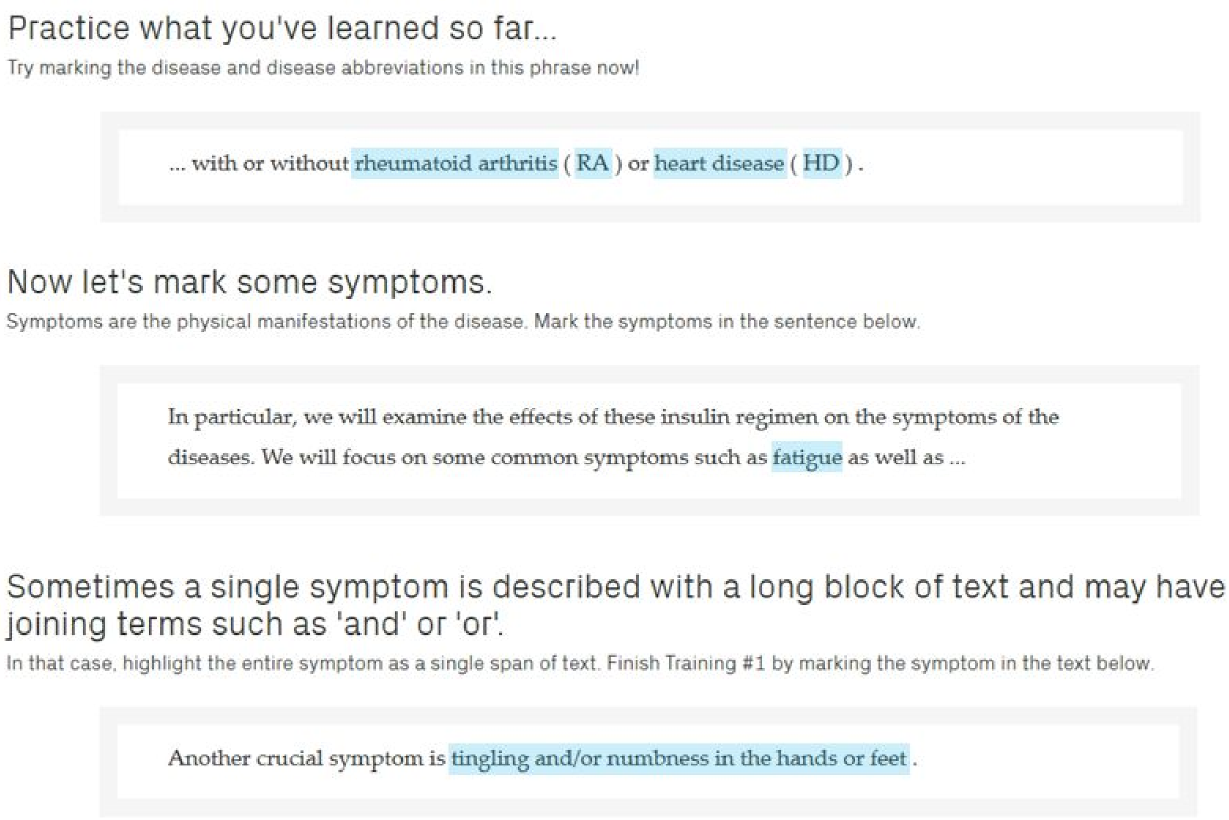

Figure S1 - Screenshots of Training 1 and the feedback screen. Training 1 introduced the user to the rules of the task in a series of nine interactive training pages and one feedback page. Each page in Training 1 generally provided one rule, a single sentence example, and an instruction on how to interact with the example in order to continue. Early user experience tests indicated that the leap from annotating single sentences (Training 1) to whole paragraphs (actual task) was daunting without some sort of transition. Training 2 acclimated the user to the task through a series of four pages with increasing amounts of text revealed on each page at a time. Training 3 introduced the feedback mechanism through a single page example.

Figure S2

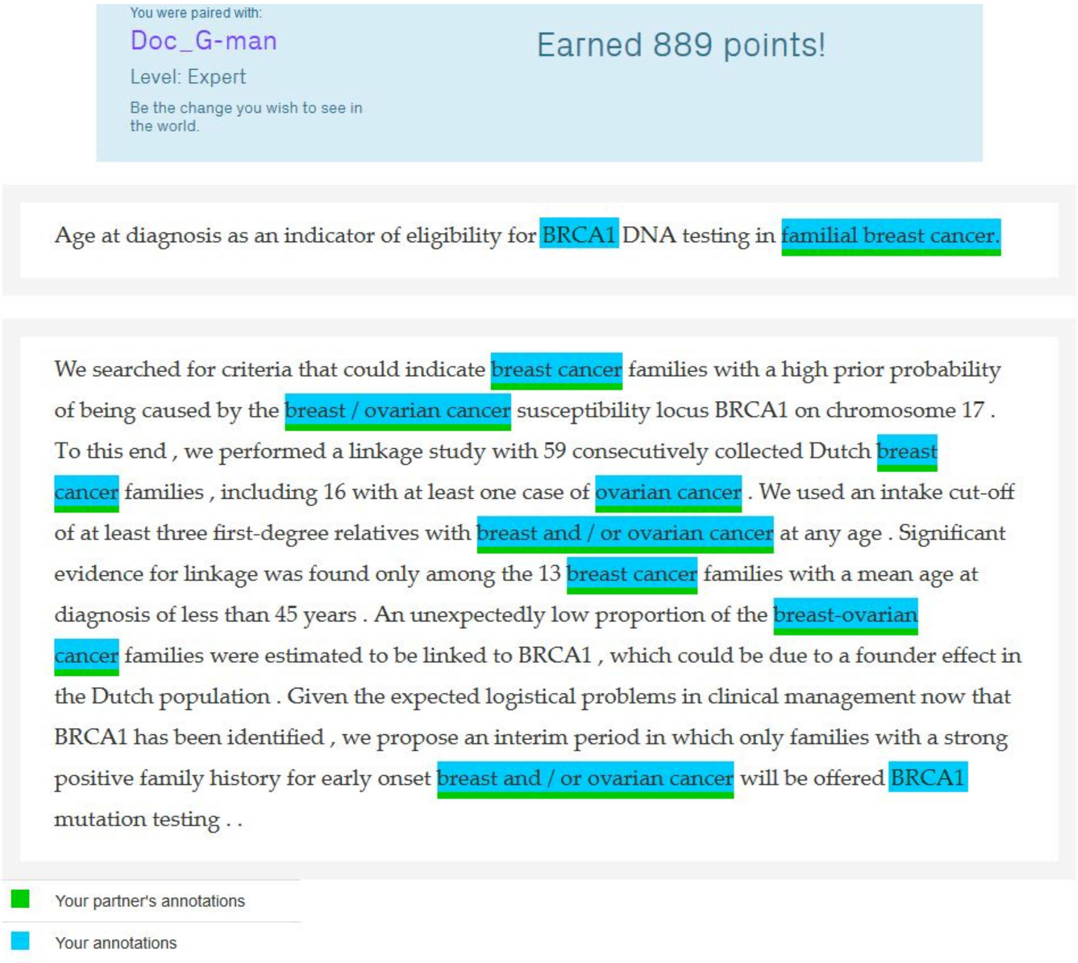

Figure S2 - Feedback screen example. A user’s annotations are compared with a partner’s in order for the user to learn. For the practice documents and gold standard documents the user is always paired with the gold standard annotations in the form of an expert partner: Doc_G-man.

Figure S3

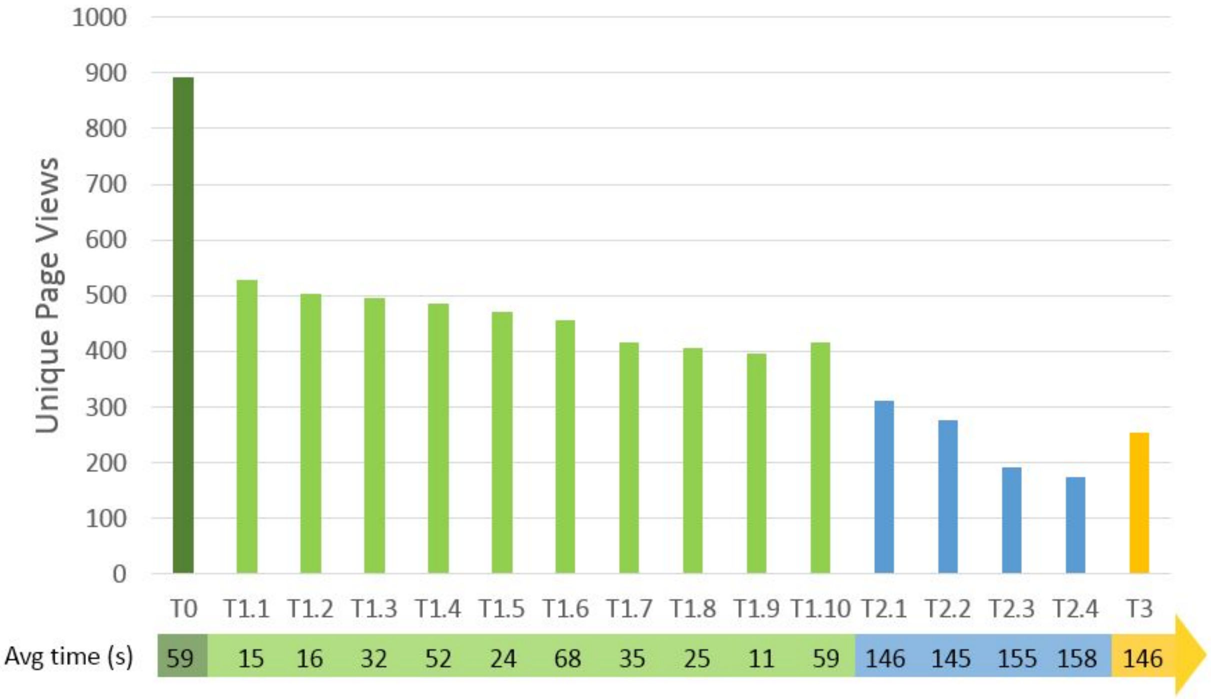

Figure S3- Page-by-page training drop off based on unique page views and average time spent per training page. Training 0 (T0) consisted of a single page with multiple available actions. Training 1 (T1) consisted of nine training pages (T1.1 - T1.9) and one feedback page (T1.19). Training 2 consisted of four training pages (T2.1 - T2.4) and Training 3 was also a single page. Unique page views increase frequently from T2.4 to T3 was likely due to the development team investigating and correcting a reported bug at that stage. Clearing the cache and revisiting that page in order to investigate the bug would increase the page view count even though it was the same user viewing that page.

The amount of time spent in Basic Training was much higher than desired, in part due to technical issues that were addressed after the experiment was started. Drop off was also high after this step as Mark2Cure was not designed for use on a mobile device, but mobile device users could progress with relative ease until Training 1.

Stepwise user drop off in Training 1 was highest between step 6 and step 7. User difficulties with step 6 was reflected in the increased average time users spent on step 6 as well as the numerous inquiries received about this step. Of the 233 emails received from participants, 22 emails specifically mentioned this step. In training 2, each step was a different abstract, and the abstracts were ordered from shortest and least complicated to longest and most complicated.

To acclimate users to seeing whole abstracts (compared to the single sentences they saw in Training 1), more and more of each abstract was revealed at each step. For example, in steps 1 and 2 of Training 2, the abstract was revealed roughly a sentence or two at a time. In step 3 of Training 2, half of the abstract was revealed at a time, and in step 4 of Training 2, the full abstract was revealed at once. Stepwise user drop off in Training 2 was highest between step 2 and step 3, which may reflect the increased difficulty of the abstract as well as the increased intimidation factor of seeing more of the abstract at once.
